# Accelerating Innovation in Health Care: Insights From a Qualitative Inquiry Into United Kingdom and United States Innovation Centers

**DOI:** 10.2196/19644

**Published:** 2020-09-25

**Authors:** Kathrin Cresswell, Robin Williams, Narath Carlile, Aziz Sheikh

**Affiliations:** 1 Usher Institute The University of Edinburgh Edinburgh United Kingdom; 2 Institute for the Study of Science, Technology and Innovation The University of Edinburgh Edinburgh United Kingdom; 3 Harvard Medical School Boston, MA United States

**Keywords:** innovation, health information technology, health care

## Abstract

**Background:**

Digital health innovations are being prioritized on international policy agendas in the hope that they will help to address the existing health system challenges.

**Objective:**

The aim of this study was to explore the setup, design, facilities, and strategic priorities of leading United Kingdom and United States health care innovation centers to identify transferable lessons for accelerating their creation and maximizing their impact.

**Methods:**

We conducted qualitative case studies consisting of semistructured, audio-recorded interviews with decision makers and center staff in 6 innovation centers. We also conducted nonparticipant observations of meetings and center tours, where we took field notes. Qualitative data were analyzed initially within and then across cases facilitated by QSR International’s NVivo software.

**Results:**

The centers had different institutional arrangements, including university-associated institutes or innovation laboratories, business accelerators or incubators, and academic health science partnership models. We conducted interviews with 34 individuals, 1 group interview with 3 participants, and observations of 4 meetings. Although the centers differed significantly in relation to their mission, structure, and governance, we observed key common characteristics. These included high-level leadership support and incentives to engage in innovation activities, a clear mission to address identified gaps within their respective organizational and health system settings, physical spaces that facilitated networking through open-door policies, flat managerial structures characterized by new organizational roles for which boundary spanning was key, and a wider innovation ecosystem that was strategically and proactively engaged with the center facilitating external partnerships.

**Conclusions:**

Although innovation in health care settings is unpredictable, we offer insights that may help those establishing innovation centers. The key in this respect is the ability to support different kinds of innovations at different stages through adequate support structures, including the development of new career pathways.

## Introduction

Health systems internationally are facing unprecedented pressures to address the challenges associated with demographic shifts while improving quality and safety and decreasing cost [1]. Digital health innovations are increasingly seen by policy makers and funders as instrumental in addressing these challenges [2-6]. Significant strategic investments are being made in this area in the United Kingdom and elsewhere, including the establishment of national innovation agencies and governmental city and regional development initiatives [7,8]. These are characterized by a range of different interpretations of the concept of innovation itself, but the majority focus on product innovation—the creation of new technological artifacts and the processes of bringing these to the market.

The creation of new technological artifacts through digital health innovation has, however, a checkered history with examples of substantial successes and dismal failures [9]. Alongside a large reservoir of potential innovations with many challenges to be addressed [10], there is a graveyard of innovations that failed completely at the outset or did not successfully scale-up [11]. This is partly due to the inherent difficulties in planning innovations in which the emergence of truly novel practice is hard to predict and technologies must satisfy a range of diverse requirements and needs [12,13]. Progress in health care innovation has been further hampered by uncertain pathways to the market, the lack of established methods for achieving success, and cumbersome processes of preparing innovations to meet exigencies of clinical governance and health service procurement [14-17].

Therefore, there is a need to better understand the innovation landscape, with a view to obtaining insights into factors that catalyze ideas and translate them into innovations that have the potential to improve outcomes for patients, providers, and health systems. There are important lessons to be learned from addressing the range of approaches adopted in different settings internationally, particularly those that have created local and regional innovation environments. These include the creation of the so-called *innovation hubs or centers*, which are collaborative, enabling spaces that bring together heterogeneous expertise from different sectors. We sought to investigate the setup, design, facilities, and strategic priorities of leading international health care innovation centers to identify transferable lessons for those seeking to accelerate or stimulate innovation within health care and identify current and common opportunities and challenges.

## Methods

### Permissions

We obtained institutional review board approval for this study from the Centre for Population Health Sciences at the University of Edinburgh, United Kingdom, on February 22, 2018. Each participant provided informed consent.

### Design

We conducted a series of qualitative case studies exploring a range of United Kingdom and United States health care innovation center facilities that are considered by funders and innovators as examples of success.

### Sampling

For our purposes, the definition of an innovation center was relatively broad as we wanted to capture the breadth of success factors across a range of contexts. Therefore, we defined an innovation center as an organizational entity that focused on incubating, developing, or accelerating new digital products for health care delivery and health promotion.

Sampling was informed by a recent mapping of leading health care innovation centers where centers that are viewed as successful are discussed [18]. The ability of centers to achieve impact and returns on investment was a key criterion to be included in our sample. We recruited centers that had been established at least 5 years before data collection (as a proxy indicator of success) to obtain insights into the challenges faced and sustainability.

To ensure maximum variation, we sampled a range of locations in the United Kingdom and the United States with a variety of foci, including academic centers, early discovery, and scale-up facilities [19]. These also included a mix of project- and product-based services and relatively new as well as established centers. Some had an emphasis on digital health products, whereas others did not exclusively focus on digital health.

Within each innovation center, we used purposive sampling to identify a diverse range of stakeholders who were involved in planning, procuring, developing, using, or managing innovation centers or associated facilities [20]. Participants comprised opinion leaders, system developers, innovators, managers, and users from various backgrounds (clinical, engineering, technology, managerial, commercial). Initial contacts were established by emailing senior innovation center leaders, and interview participants were snowball sampled through these contacts.

### Data Collection

Data collection in each case study consisted of semistructured, in-depth one-to-one interviews or, if more convenient for interviewees, telephone interviews. Interviews were conducted with a topic guide (Textbox 1), exploring views on the setup, culture, and features of innovation approaches as well as expected and experienced benefits, experiences and lessons learned, perceived challenges, and potentially transferable lessons. Questions were informed by conceptual work led by one of our coauthors (AS) scoping where and how innovations in health care settings are succeeding [18].

All interviews and site visits were conducted by the same researcher (KC). Interviews were, with permission, digitally audio-recorded. However, audio-recording was not feasible in 5 interviews because these took place in noisy environments. In such instances, the researcher took extensive field notes. Recordings were then transcribed verbatim together with accompanying field notes.

We were opportunistic in developing our program of visits and observations, with the researcher joining center meetings and guided tours of physical spaces. At these, the researcher took field notes that were unstructured but involved recording the location, people, the topic of discussions, impressions on the environment, and any other emerging impressions on social dynamics.

Data generation ended when saturation was reached and no significantly new themes emerged from the concurrent data analysis [21].

**A sample topic guide.**

Interviewee’s background: current position, role in relation to the innovation center

Setup and facilities (technologies, networks, and managerial structures)

Expected and experienced benefits

Facilitators for and barriers to health care innovation

Challenges and lessons learned

Sustainability models

Anything else?

Anyone else we can speak to?

### Data Analysis

Qualitative data collection and analysis were iterative, allowing emerging themes to be explored further while seeking disconfirming evidence [22]. Coding was informed by our extensive previous literature reviews on digital health interventions and earlier work on innovation environments [23,24]. Our coding framework was based on this extensive previous work, providing an overall initial coding structure. We did, however, also allow new themes to emerge and refined the framework accordingly. These new dimensions are discussed in detail in the Results section.

Interview notes, transcripts, and observation notes were uploaded onto the NVivo11 software and initially coded against the topic guide categories by case study (within-case analysis). As data analysis progressed, we identified new categories and rearranged codes and subcodes to present a holistic picture of innovation center strategies, stakeholders, and environments. In doing so, we combined a diverse range of interviewees, perspectives, facilities, and contexts. Detailed within-case analysis was followed by analysis across cases to identify overarching themes, similarities and differences between cases, and potential implications for other settings.

## Results

We visited 6 international innovation centers in the United States and the United Kingdom, conducted interviews with 34 individuals and 1 group interview with 3 participants, and observed 4 center meetings. The characteristics of the centers are provided in Table 1, and the characteristics of the interviewees are provided in Table 2.

**Figure 1:**
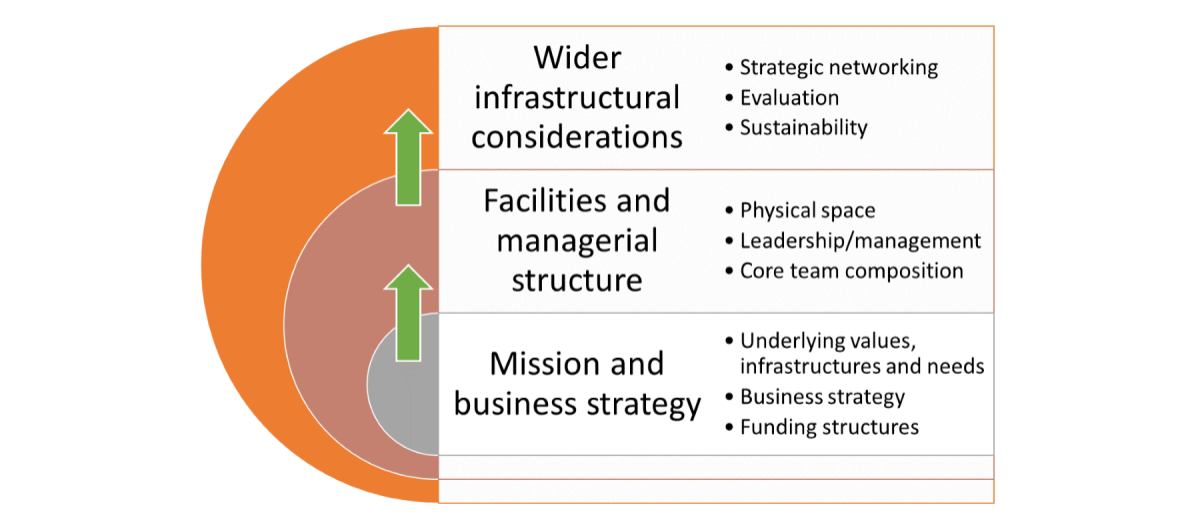
Overview of findings.

All the centers had different physical and organizational setups and facilities, depending on their primary purpose. However, we also identified some common threads across the settings. Overarching themes and subthemes are illustrated in Figure 1.

**Table 1:** Innovation center characteristics.

Center number	Primary purpose	High-level overview	Date established; how they started	Ongoing funding model	Outputs	Location
1	Four innovation centers with focus on drug development, innovation incubation, and research acceleration part of 1 university umbrella organization	Located on the university campus, some degree of networking across centers	2000, 2006, 2010, 2011	Commercial funding University funding Fellowship and teaching model	Technological products Education Networking	United States
2	Rapid start-up facilities for commercial companies and digital creative industries	Managed office space specializing in incubating digital health companies	2013; designed to promote growth for high-growth tech start-ups	Tenancy (letting out space to companies)	Technology acceleration and scale-up	United Kingdom
3	Corporate accelerator, variety of health-related specialties	University-affiliated, focus on evidence-based innovation	2009; to identify, develop, and scale evidence-based health care innovation	Funding councils University (operational budget) Commercial Philanthropy	Technology acceleration and scale-up	United Kingdom
4	Academic health science partnership	Virtual network consisting of scientists, health care staff and organizations, support to establish relationships	2009; to translate research and innovation into benefits for patients and populations	Funding councils	Education and networking leading to digital innovation	United Kingdom
5	Coworking space	Managed office space specializing in establishing partnerships through coworking	2010; to create workspaces that help to create communities of practice	Tenancy (letting out space to companies)	Relationship building leading to digital innovation	United Kingdom
6	Digital health start-up center	Located on hospital premises, focus on helping stakeholders to find out if their product is (or could be made) commercially viable and provide support for the development process	2013; to drive internal innovation	Health care organization operational budget	Technologies that can be used in the operational hospital environment	United States

**Table 2:** Interviewee characteristics.

Participant number	Gender	Occupation and role in the center	Center number	Location
1	Male	Director	2	United Kingdom
2	Male	Designer	3	United Kingdom
3	Group interview (1 male, 2 females)	Director, 2 innovation managers	4	United Kingdom
4	Male	Director	3	United Kingdom
5	Male	Director	2	United Kingdom
6	Male	Advisor	3	United Kingdom
7	Male	Clinician	3	United Kingdom
8	Male	Innovation champion	3	United Kingdom
9	Female	Clinician	2	United Kingdom
10	Female	Strategy consultant	2	United Kingdom
11	Male	Academic	3	United Kingdom
12	Male	Innovation manager	6	United States
13	Male	Innovation analyst	6	United States
14	Female	Innovation manager	6	United States
15	Male	Clinician	6	United States
16	Male	Clinician	6	United States
17	Male	Engineer/designer	6	United States
18	Male	Director	6	United States
19	Female	Academic	6	United States
20	Male	Policy	6	United States
21	Male	Academic/clinician	6	United States
22	Male	Industry	6	United States
23	Male	Academic/clinician	6	United States
24	Male	Academic/clinician	6	United States
25	Male	Academic/clinician	6	United States
26	Male	Academic/clinician	1	United States
27	Male	Academic/clinician/director	1	United States
28	Male	Academic/clinician	1	United States
29	Male	Academic	1	United States
30	Female	Academic/director	1	United States
31	Female	Academic/codirector	1	United States
32	Male	Director	1	United States
33	Male	Academic/clinician/director	1	United States
34	Male	Academic/clinician	1	United States

### Mission and Business Strategy

Different centers had various underlying values, infrastructures, and needs. However, all worked hard to create organizational cultures that placed innovation at the core of their activities. This involved actively engaging with external communities to promote shared knowledge (what has worked commercially and what the important problems within health care are) and the creation of easier pathways for opportunities to solve those problems:

Just generally just to have a buzz going on about innovation is happening and what’s going on, and just bringing promotion internally as well as externally, so that we’re open, the external community knows, like we are open for business.

Participant 12, male, innovation manager, United States

Common to all was also an effort to align with the various key local and national societal and health system challenges and existing technological and social infrastructures, and bring together various stakeholders involved in these. Centers had portfolios that combined more and less adventurous or disruptive innovation forms, allowing to focus on solving the most pressing real-world problems while still satisfying market needs and coordinating with other existing initiatives. The key here was perceived to be the alignment of commercial, clinical, and patient needs and values, as these could act as incentives for the various stakeholders who need to be involved:

…the product has to address a significant medical need, number one...the majority of times, that’s going to require that it’s going to be commercially attractive, right?

Participant 29, male, academic, United States

Defining a unique proposition to adopters that was not addressed elsewhere was seen as essential to create value and impact:

You’ve got to be very clear what it is that you’re trying to do in terms of establishing a unique selling point and a unique position within the market.

Participant 6, male, advisor, United Kingdom

Activities frequently involved mapping key local, national, and international stakeholders of potential relevance to the center and aligning their motivations, values, and needs with activities. For example, there was often a focus on bringing together *communities of interest*, such as commercial sectors, academic settings, and health care professionals, thereby bridging the gap between the problem, idea, product, and use of the product in context.

To ensure value to patients, it was argued that the first stage of the innovation process should be to identify existing needs and thereafter identify the technology that might address those needs. This needs-based approach was also seen to help bring different stakeholders together as a fundamental starting point for further activities that facilitated aligning different viewpoints and incentive structures around clinical, patient, and economic needs:

…if you’re trying to start with a technology and then hoping that what you come up with is going to be received positively, by all these stakeholders, your chances are pretty low. But if you can start with...understanding the stakeholder landscape, in the beginning then you have a better chance.

Participant 32, male, director, United States

Although stakeholders often had different expectations and needs in relation to timelines, which required a great deal of relationship building, the centers frequently took on this intermediary role and acted as connectors of otherwise disconnected worlds (although these again varied across the centers they included, for instance, academic health care and commercial domains):

I think our role has been making sure they get connected with the people who can help them, and so expanding across the network was not just them, they could not have done that by themselves.

Participant 15, male, clinician, United States

To be commercially viable, innovation center leadership had to balance a number of tensions. These included aligning organizational priorities with stakeholder motivations (which may both be subject to change over time), senior leadership commitment to innovation while allowing a degree of local creativity, and some risk taking (eg, funding for *risky projects* while ensuring a steady stream of income):

...we have that appetite to take on a little bit of risk to work with these newer companies as long as there’s alignment and we know that it’s a good team, it’s a good product, it’s something that we will derive value from.

Participant 18, male, director, United States

Most centers had a mixture of short-term *risky* funding sources they had won or could give out (eg, seed funding, which was particularly relevant for early development and proof of concept of innovations) and also relied on more stable sources of income for security (eg, operational or research council funds). More stable funding was often associated with sustained investment to bring innovations to a point where they could survive in the market. Textbox 2 summarizes the various funding sources discussed by the participants.

**Summary of funding sources.**

Seed funding for start-up

Organizational operations budget (to ensure alignment with operational objectives)

Grant funding (government, research councils)

Commercial funding (eg, venture capital firms)

Private foundations (eg, angel investors, philanthropic donations)

Tenancy (letting out space to companies)

Fellowship and teaching funding

### Facilities and Managerial Structures

Physical space and buildings varied from one-room office spaces to whole buildings, where center staff were colocated with commercial companies, and centers that were located within a health care organization. A crucial feature of many centers was an *open-door policy*, meaning those with ideas who wanted to innovate could come in as a first point of contact:

Anyone...can come, schedule some time with one of our members and talk about and hash through the idea of where they are, what the next step is.

Participant 16, male, clinician, United States

If clinicians were identified as important stakeholders (which depended on the primary purpose of the center), colocation with clinical premises was important so that clinicians could make use of the facilities, attend events, and network.

Another important characteristic of the physical space was flexibility such that facilities could be used for multiple purposes (including individual working, hosting meetings, external events, and conferences). Spaces also promoted colocation and were adaptable to ensure responsiveness to changing stakeholders’ needs over time. If centers consisted of whole buildings, these encouraged social contacts on an informal basis and provided ample opportunities for people to meet either in a planned manner or opportunistically, including cafes and pleasant outside spaces. In short, most spaces consisted of an enriching and engaging environment that staff wanted to spend time in and that they were proud to show off to external stakeholders:

Why this building is a very important investment for the hospital and, you know, why people are super-excited about it because basically for the first time you would have clinical and research right here where people could easily bump into each other and talk to each other.

Participant 19, female, academic, United States

The director of a center told us that “*the single two most important things are good Internet and good coffee*” (note, center visit).

Leadership often comprised a small, tight-knit team from diverse backgrounds, with commercial, managerial, and clinical knowledge, networks, and skills that aligned with the purpose and mission of the center, combined with an in-depth understanding of the organizational environment (although not all centers were embedded within health care institutions):

You have to get people who understand where the bodies are buried in the system you’re trying to disrupt.

Participant 1, male, director, United Kingdom

Management structures tended to be relatively flat and informal, with strong high-level leadership support for innovation while still allowing team members a degree of creative flexibility. This meant that innovation centers were in some cases not bound by institutional regulations or tied to hierarchical structures that might limit new ways of working and building relationships. For instance, academic and clinical members were often relieved of some pressures associated with their other roles by means of secondments or protected time to innovate:

...I think it depends on willingness and whether the environment is conductive to do this. I mean, what would the university say if a professor says that, I want to spend x per cent of my time on this, for various values of x. Would they be sympathetic? Would they be questioning? Would they be outright negative? Do they allow someone to take big chunks of their time to leave for six months, one year, two years...and build something and then leave it to some other team and come back to the university?

Participant 3, male, academic/clinician, United States

Team attitude and culture seemed to play a particularly important role with a common drive to *get things done*, a risk-taking attitude, and a focus on collectively solving problems. Staff members in many cases had an intrinsic commitment to innovation with a strong belief that this was the right way forward to solve health care challenges. Center teams were often proactively recruited by senior leadership. Here, skills and interpersonal capabilities were important:

I would say probably more a willingness to be able to talk to others and that personality to be a connector. That’s more important...you saw it at the Apple Store and they didn’t care that you had any Apple products or if you knew anything about technology. Those are things that they can teach you. But they care more about your people skills and then your personalities because that’s very hard to teach...

Participant 13, male, innovation analyst, United States

Although certain characteristics were common (eg, managerial backgrounds), we observed a different combination of professional skills and backgrounds, depending on the primary objectives of the center. Many core staff members had experience working in different settings (eg, commercial, academic, clinical, managerial) and the ability to span boundaries, move between different worlds, and connect them (eg, entrepreneurial researchers or highly research-minded entrepreneurs).

The core staff tended to work strategically with third parties where specific skills or specialties needed to be brought in to support different aspects of the innovation journey. Creatively drawing on these was viewed as crucial.

The emergence of innovation centers as a relatively new development also demanded the creation of hybrid roles such as innovation strategy managers and innovation analysts who had no established career pathways and may therefore struggle to prove their value to the wider organizational setting:

...innovation managers, the people there to understand and think about describing their own value to their organisations and career pathing for the people here, for the innovation managers here and across all of the innovation team…hospitals don’t really know how to value these people. The people in those roles often don’t have a language to describe what they do and the value they bring to their institution, to researchers and so on.

Participant 20, male, policy, United States

### Wider Infrastructural Considerations

Strategic networking with external stakeholders was a key activity across all centers we visited, frequently characterized by proactive efforts to build relationships with collaborators. These varied across centers, depending on the core mission, but often included academic institutions, policy makers, health care providers, patient organizations, current and future funders, and commercial organizations. In doing so, centers brought together stakeholders who would not typically meet in intensive time-limited interactions and events (such as accelerators, hackathons, challenges, conferences, sandbox events, and competitions):

So I think the events serve as a major platform for bringing people together and there are some events that we’ve done specifically targeting those kinds of, you know, interactions.

Participant 19, female, academic, United States

Publicizing interactions and events was key to these interactions with dedicated public relations support to promote positive messages and celebrate individual and group wins.

Most acknowledged that there was a critical need to align activities with the wider innovation ecosystem. Centers that were part of this study had placed themselves at the center of academic, commercial, and governmental networks. They were strategically placed in attractive cities that were easily reached through national and international travel networks. A vibrant commercial environment featured heavily and was purposefully aligned to leading universities and health care organizations, where relevant. In some instances, this created a fluid talent pool of people moving between sectors and bridging multiple communities:

There’s probably less of that here because a lot of people will switch careers back and forth all the time, so the talent pool is pretty fluid. So you’ll have a lot of people that will cross-pollinate between the different groups. So I think some of the culture tends to merge a little bit.

Participant 17, male, engineer/designer, United States

As such, the ecosystem became somewhat of a magnet that drew entrepreneurial spirits in and attracted a certain type of person, which, in turn, was seen to transform the ecosystem:

...everybody from everywhere wants to come here to make their money and be where the excitement is.

Participant 33, male, academic/clinician/director, United States

However, the mismatching timelines of different institutional stakeholders were frequently cited as barriers to innovation, with commercial partners needing to move quickly and academic and health care settings being averse to risk and therefore less equipped for moving fast owing to often deeply engrained bureaucratic procedures and hierarchical structures.

In addition, despite a general recognition of the importance of evaluation, many centers struggled with establishing measurable metrics that indicated value. Financial metrics are important for all types of organizations. Although start-ups, accelerators, and incubation centers tended to focus on the number of patents and companies created, university-affiliated centers tended to focus on improvements in the quality and safety of care and staff and patient experience.

A further challenge consisted of aggregating project metrics to make claims about the overall success of the center:

When we’re talking about how we measure the programme as a whole something we’ve wrestled with is how do we then take all these disparate clinical metrics and make them into something you can come up with. Because we’ve tried to sell the programme as we’re innovating, we’re delivering better healthcare and we’re delivering financial value.

Participant 15, male, clinician, United States

Many also struggled to allocate appropriate time and resources to investigate *failed* initiatives, although the importance of this activity was generally recognized:

...when we fail, we tell everybody...that we failed because there is so much more learning from that...

Participant 30, female, academic/director, United States

Another challenge was scaling, as it was perceived as one of the most unpredictable aspects of the innovation journey and required dedicated resources and expertise. Therefore, some stated there was a danger that innovation centers would “*create a thousand different solutions that the system does not want*” [Participant 5, male, director, United Kingdom].

## Discussion

### Summary of Findings

We collected qualitative data from a range of settings and identified some important common characteristics of the way different innovation centers approached innovation. Successful centers brought together various combinations of expertise and experience (including academic, technology, service delivery, professional, business, and regulatory) to promote innovations. In the context of myriad opportunities, they helped to identify pain points, stimulate ideas, and facilitate the development of promising avenues. They did so by traversing key segments of the innovation journey, from early high-risk, unproven potential to commercial investment appraisal.

### Strengths and Limitations

Although efforts have been made to characterize features of particular successful innovation environments, attempts to reproduce these *critical success factors* in other settings have mostly failed to deliver, as outcomes are unpredictable and contingent on particular forms and contexts of innovation [25]. Our study provides a starting point for those wishing to navigate this challenging area, providing insights into stimulating innovation in high-risk health care environments.

However, innovation research and policy have been held back by a lack of agreed definitions of innovation, with many efforts and centers focusing on product innovation. Therefore, our empirical focus was somewhat limited, potentially neglecting other types of innovation (eg, organizational, service, and social innovation) and different innovation pathways [26-30].

This was an exploratory study that focused on United Kingdom and United States settings, as we wanted to establish factors that have been identified to promote innovation across contexts. Limitations include the modest number of cases and variations in the data collected within each center. Ideally, we would have sampled a wider range of centers in different geographical locations, including other countries, and with a similar focus to produce more comparable results. We would also have liked to recruit a more comparable sample of individual respondents (including a greater number of *on-the-ground* staff) and a more balanced representation of men and women (although this may reflect innovation center workforce trends), but we were limited by accessibility to centers and individuals. We also had to navigate complex approval procedures and a certain degree of trade-off between the depth and breadth of data collected.

In addition, this was a retrospective study of stakeholder perceptions; therefore, it will likely be subject to recall bias. Successful innovations are those in which various possible barriers were avoided and challenges negotiated along the way—a long-term process that is highly unpredictable and is therefore difficult to extrapolate inductively from one case to the next. Similarly, there are likely to be different support models depending on the stage of innovation and the age, size, and funding model of the center. For example, some factors, such as the ability to take risks, may be less realistic in smaller and less well-funded centers, and these centers may also have smaller teams and more restrictive environments than large well-funded centers (eg, old buildings that do not allow for colocation). It may be that in these environments other factors take more prominent roles than others. For example, if the building is not suitable for colocation, then staff may need to compensate by moving around and actively network within the wider ecosystem. There may also be the need to identify other factors that help a center stand out, for example, including a broad range of stakeholders in their activities such as patient and public representatives. Different stages and centers will likely require different roles and foci [31]. Longitudinal real-time ethnographic studies could help to address these issues. These should also seek to identify which innovation initiatives are most likely to be *successful* under what circumstances and develop a quantifiable set of indicators to guide future efforts.

### Integration of Findings With the Current Literature

We observed some seemingly paradoxical requirements surrounding the support structures. On the one hand, there was a need for frameworks that encouraged diversity to promote ideas at the outset (eg, where different interdisciplinary groups could meet in short-duration projects). On the other hand, there was a strategic requirement to focus on sustained investment in particular areas to bring innovation to the point it could survive in the market [32]. This strategy seems appropriate for navigating uncertainty, given that many promising innovations are likely to fail along the journey [33].

Our study also shows that there is a clear need for new career pathways for organizations to support different forms of innovation and the need to develop roles and skills for different stages of the innovation journey. These include new hybrid roles of boundary spanners (connectors) who can bridge different worlds. Interpersonal skills and previous experience in context are key in this respect. In addition, successful innovators are often those who have been previously involved in a series of earlier *failed* innovations through which they have gained the resources, reputation, experience, knowledge, and linkages needed for eventual *success* [34].

Health care organizations could promote the exploitation of these skills through the creation of career pathways around new kinds of hybrid roles. For instance, staff need to be presented with opportunities to enhance their capabilities, knowledge, and links with multiple arenas through their involvement in a series of projects. Innovation calls for access to diverse skills, which may include engineering, venture capitalism, entrepreneurship, financial services, investment communities, technology companies, academia, and clinical and professional services. Key intermediaries often internalize understanding or links to these kinds of expertise.

Both countries in which we carried out our research had firmly established national digital health system strategies, and these national drives resulted in national funding for related innovation, research, and innovation from which the centers benefited. However, there was a marked difference in vendor landscapes and health system competition (public sector vs competitive insurance market), which are likely to have impacted various innovation and acceleration efforts. For example, there may have been stronger drivers for economic gains and a stronger pool of professional expertise driven by the competitive insurance market.

### Implications for Practice and Further Research Emerging From This Study

On the basis of this study, we developed a framework of key considerations that a leadership team may wish to consider when establishing a health care innovation center and revising short-, medium-, and long-term strategies. These are summarized in Table 3. This list is not intended to be exhaustive, and dimensions are likely to vary across localities. It should therefore be seen as a guide to structure thinking and not as a recipe for success.

There are also some important implications for informaticians. The key here will be establishing a needs-based approach to innovation that is driven by engaging a range of stakeholder communities and aligning their values. This may be achieved by exploring how different needs can be addressed through various forms of innovation before developing new technological artifacts.

**Table 3:** Key considerations when establishing and guiding a health care innovation center.

Priority weighing	Area	Key consideration
Priority 1	Need and opportunity	Alignment with key local and national societal and health system challenges
Priority 2	Leadership	Senior-level commitment to innovation and associated commitment to associated organizational changes
Priority 3	Strategic prioritization and incentives	Alignment of the center with organizational priorities in the short, medium, and long term and associated incentives
Priority 4	People	Valuing and promoting interpersonal skills and boundary-spanning capabilities
Priority 5	Culture	Flat and informal management structures allowing a degree of creative flexibility
Priority 6	Funding and resources	Diversity in funding sources allowing a mixture of stable and *risky* investments
Priority 7	Relationships	Allowing the organic development of communities of practice around specific challenges
Priority 8	Space	Easily accessible, pleasant, flexible, and conducive to formal and informal networking by a variety of parties

### Conclusions

Although definite measures of success are difficult to establish, we have begun extracting some key considerations that can be used by planners and implementers to guide the establishment and maintenance of health care innovation centers. These include strategy and leadership that view innovation as an organizational priority, establishing organizational cultures and structures that allow experimentation and creative flexibility, and designing physical environments that facilitate networking and relationship building.

There is a clear need to consider different forms of innovation and how these require different kinds of organizational support structures, including establishing new career pathways for hybrid boundary spanners.
